# Stratification of Acute Kidney Injury in COVID-19

**DOI:** 10.4269/ajtmh.20-0794

**Published:** 2020-10-27

**Authors:** Tauqeer Hussain Mallhi, Yusra Habib Khan, Azreen Syazril Adnan

**Affiliations:** 1 Department of Clinical Pharmacy, College of Pharmacy, Jouf University, Sakaka, Kingdom of Saudi Arabia;; 2 Department of Nephrology, MSU Medical Centre, Management and Science University Shah Alam, Malaysia;; 3 Chronic Kidney Disease Resource Center, School of Medical Sciences, Hospital Universiti Sains Malaysia, Kota Bharu, Malaysia

## Abstract

Despite myriad improvements in the care of COVID-19 patients, atypical manifestations are least appreciated during the current pandemic. Because COVID-19 is primarily manifesting as an acute respiratory illness with interstitial and alveolar pneumonia, the possibility of viral invasions into the other organs cannot be disregarded. Acute kidney injury (AKI) has been associated with various viral infections including dengue, chikungunya, Zika, and HIV. The prevalence and risks of AKI during the course of COVID-19 have been described in few studies. However, the existing literature demonstrate great disparity across findings amid variations in methodology and population. This article underscores the propensity of AKI among COVID-19 patients, limitations of the exiting evidence, and importance of timely identification during the case management. The prevalence of AKI is variable across the studies ranging from 4.7% to 81%. Evidence suggest old age, comorbidities, ventilator support, use of vasopressors, black race, severe infection, and elevated levels of baseline serum creatinine and d-dimers are independent risk factors of COVID-19 associated with AKI. COVID-19 patients with AKI also showed unsatisfactory renal recovery and higher mortality rate as compared with patients without AKI. These findings underscore that AKI frequently occurs during the course of COVID-19 infection and requires early stratification and management.

Recent research advances on COVID-19 have explicitly underscored the classical respiratory symptoms associated with the disease. However, the growing body of evidence describes numerous atypical manifestations in association with the disease course. Acute kidney injury (AKI) is recently emerged as a potential complication during the course of COVID-19.^1^ However, current investigations demonstrate wide disparities in the prevalence and mechanisms of COVID-19 associated with AKI. A recent case series by Wang et al.^2^ reported that AKI is uncommon among COVID-19 patients and even not seen in preexisting chronic kidney disease (CKD). Given the differences across findings, we felt inclined to share our point of view regarding the propensity of AKI during the course of COVID-19. We believe that odds of AKI occurrence among COVID-19 patients are substantially higher and must be considered during the case management. Because of unprecedented COVID-19 cases, clinicians are primarily engaged in the case management along with virus containment to avoid the disease spillover, and there is high propensity that healthcare professionals may overlook a slight rise in serum creatinine (SCr) among patients.

Because COVID-19 is primarily manifested as an acute respiratory illness with interstitial and alveolar pneumonia, the possibility of viral invasions into other organs cannot be disregarded. It is pertinent to mention that AKI remained neglected intricacy during various viral infections and was later emerged as highly morbid and fatal complication.^3,4^ Dengue-associated AKI is such an example which was initially least appreciated during various outbreaks and was subsequently evolved as a serious complication.^5^ Although Wang and others did not observe any substantial rise in SCr among patients, a firm conclusion cannot be drawn amid small sample size (*N* = 116) in their study.^2^ Recent investigations accentuate the involvement of multiple organs in COVID-19^6,7^ and report AKI in 6–36.6% of cases.^8–10^ Subsequent studies reported that COVID-19 presents with massive albuminuria (34%), elevated blood urea nitrogen (27%), proteinuria along with hematuria (44%), and increased SCr (15.5%),^11–13^ either on admission and or during hospitalization. A Chinese study indicated abnormal urine dipstick in 75.4% hospitalized patients.^14^ A large cohort study from New York showed that 52.2% patients receiving mechanical ventilation developed AKI within 24 hours following intubation, suggesting mechanical ventilation as a significant predictor of AKI.^10^ Furthermore, serious concerns have also been raised about the impending shortage of renal replacement therapy for COVID-19 patients.^15^ Moreover, AKI has also been estimated as an independent predictor of in-hospital mortality among COVID-19 patients.^13,16^ On the other hand, a Japanese study showed that COVID-19 portends higher mortality (16.2%) among dialysis patients than the general population (5.3%), suggesting hospitalization of these patients.^1^ Table 1 describes the descriptive summary of studies illustrating renal involvement during the course of COVID-19.

**Table 1:** Summary of studies describing the renal abnormalities and prevalence of AKI during COVID-19 infection

Authors, year	Country	Sample size	Demographics	Type of renal abnormalities	Prevalence of AKI	Outcomes/remarks
Chen et al.^25^	China	*N* = 274 (dead: 113, recovered: 161)	Median age: 60 years, 73% males	AKI (11%), proteinuria (60%), hematuria (50.6%)	AKI in total cohort: 11%	The renal anomalies were common in dead cases as compared with recovered patients
					AKI in died cases: 25%	
					AKI in recovered cases: 1%	
Cheng et al.^11^	China	*N* = 701	Median age: 63 years, 52.4% males	OA elevated SCr (14.4%), OA elevated BUN (13.1%), glomerular filtration rate ≪ 60 mL/minute per 1.73 m^2^ (13.1%), proteinuria (43.9%), hematuria (26.7%)	AKI: 5.1% during hospital stay; prevalence of AKI was higher among patients with elevated SCr on hospital admission	Elevated SCr, BUN, hematuria, proteinuria, and AKI (stage 2) were associated with mortality.
Pei et al.^14^	China	*N* = 333	Mean age: 56.3 years, 54.7% males	Overall renal involvements (75.4%), proteinuria (65.8%), hematuria (41.7%)	4.7% by KDIGO criteria and 7.5% by expanded KDIGO criteria	Overall mortality: 29 (20 cases had AKI defined by either criteria), out of 16/35 AKI cases (defined by expanded criteria) showed renal recovery
Taher et al.^24^	Bahrain	*N* = 73	Mean age: 54 years, 60.3% males	Hematuria (20.5%), proteinuria (52.1%)	AKI: 29/73, 39.7% (stage 1: 11%, stage 2: 15.1%, stage 3: 13.7%)	7 cases required RRT, 12/13 died cases had AKI, renal recovery was observed in 16 cases, one patient was discharged on dialysis
Hirsch et al.^10^	USA	*N* = 5,449	Median age: 64 years, 60.9% males	Hematuria (36.5%), leukocytouria (40.9%), protein 3+ (78 cases)	AKI: 1993/5,449, 36.6% (stage 1: 46.5%, stage 2: 22.4%, stage 3: 31.1%)	AKI was substantially associated with mortality, risk factors of AKI included were old age, cardiovascular disease and mechanical ventilation
Joseph et al.^22^	France	*N* = 100	Median age: 59 years, 70% males	Only AKI	AKI: 81/100, 81% (stage 1: 44 cases, stage 2: 10 cases, stage 3: 27 cases)	Overall 29 death cases, 28 cases had AKI
Ng et al.^26^	USA	*N* = 9,657	Median age: 62 years, 58% males	Only AKI	AKI: 3,854/9,657	Among AKI patients without RRT, 74% showed renal recovery at discharge,
					(3,116 cases had non-kidney replacement therapy required AKI, 638 cases had stage 3 AKI requiring replacement therapy)	Among patients with AKI requiring replacement therapy, 30.6% remained on dialysis on discharge
Cui et al.^23^	China	*N* = 116	Mean age: 59 years, 56.9% males	Only AKI	AKI: 21/116, 18.1%	Overall mortality rate was 15.5%, patients with AKI had higher mortality rate than those without AKI
					Early AKI (developed within 72 hours): 13 cases	
					Late AKI (developed after 72 hours of admission): eight cases	

AKI = acute kidney injury; BUN = blood urea nitrogen; SCr = serum creatinine; KDIGO = kidney disease: improving global outcomes; RRT = renal replacement therapy.

The renal injury during the course of COVID-19 is multifaceted, involving various triggers. Renal hypoperfusion–related acute tubular necrosis or cellular damage, dysregulated inflammatory response, microcirculatory dysfunction, metabolic reprogramming, cytokine storm syndrome precipitated by sepsis, and direct viral injury are the possible etiopathological mechanisms (Figure 1).^13^ Moreover, the presence of angiotensin-converting enzyme 2 receptors in renal tissues could also facilitate viral invasion, resulting in direct injury.^17,18^ Viral RNA has been isolated from the urine sample of one patient (6.9% of 58 cases), suggesting kidney as a vulnerable target for infection.^19^ Viral particles have also been detected in the postmortem kidney samples of COVID-19 fatal cases.^20^ These findings underscore the capability of the virus to cause renal damage either by direct invasion into the renal parenchyma or through other secondary mechanisms.

**Figure 1.**
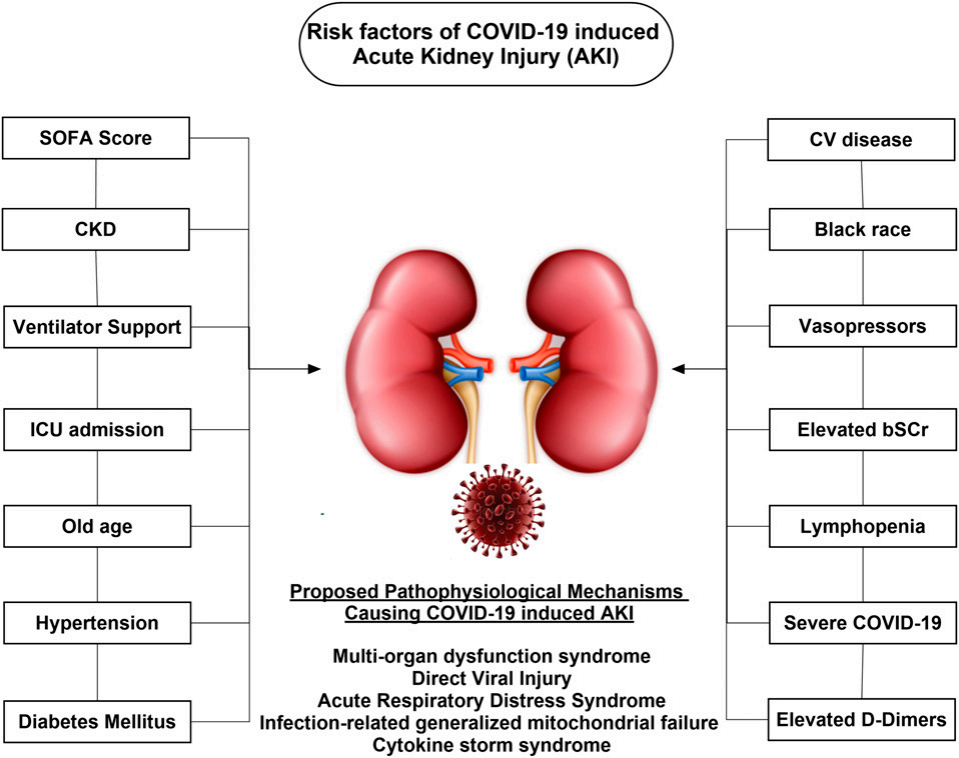
Risk factors for the development of acute kidney injury (AKI) during COVID-19 infection.

Some additional studies suggest association of COVID-19–induced AKI with advance age and preexisting comorbidities.^21^ Available studies^10,11,22–24^ have reported various risk factors for the development of AKI during the course of COVID-19 as described in Figure 1. Taken together, in contrast to the results of Wang et al.,^2^ these findings underscore that AKI is frequently encountered^10^ highly morbid and fatal complication^10,14,22,23^ during COVID-19 and warrant the dire need of nephrology consultation. However, these studies are accompanied by potential limitations including the less number of patients, unavailability of baseline SCr, disparity in AKI diagnostic criteria, and lack of follow-up to ascertain the renal recovery. Future studies must be conducted in light of these limitations, so findings could be appropriately incorporated into the clinical practice. The use of baseline SCr is subjected to wide variations and depends on the individual clinical settings. Moreover, diagnostic criteria of AKI must be followed to ascertain the true estimate. Clinicians must adhere to the kidney disease: improving global outcomes criteria on classification and treatment of AKI to establish the uniformity across the literature.

Before the era of CKD staging, AKI was considered as reversible with satisfactory renal outcomes. However, with the recent advancements in renal care, it is widely accepted that patients who survive an episode of AKI might recover adequate renal functions, but still such patients are at risk of developing CKD.^3^ Considering the widespread nature of the disease and substantial number of patients with atypical manifestations, subsequent renal deterioration following an episode of AKI may pose substantial risks of CKD among survivors. We urge early stratification of AKI during COVID-19 case management which would provide opportunity to clinicians for appropriate management in a timely manner. Moreover, the follow-up of patients to ascertain the renal recovery will be of paramount importance to design and implement intervention strategies for high-risk patients.
